# Induced *Burkholderia* prophages detected from the hemoculture: a biomarker for *Burkholderia pseudomallei* infection

**DOI:** 10.3389/fmicb.2024.1361121

**Published:** 2024-04-02

**Authors:** Patoo Withatanung, Sujintana Janesomboon, Muthita Vanaporn, Veerachat Muangsombut, Sorujsiri Charoensudjai, Dave J. Baker, Vanaporn Wuthiekanun, Edouard E. Galyov, Martha R. J. Clokie, Ozan Gundogdu, Sunee Korbsrisate

**Affiliations:** ^1^Department of Immunology, Faculty of Medicine Siriraj Hospital, Mahidol University, Bangkok, Thailand; ^2^Department of Microbiology and Immunology, Faculty of Tropical Medicine, Mahidol University, Bangkok, Thailand; ^3^Department of Microbiology, Faculty of Medicine, Khon Kaen University, Khon Kaen, Thailand; ^4^Science Operations, Quadram Institute Bioscience, Norwich, United Kingdom; ^5^Mahidol-Oxford Tropical Medicine Research Unit, Mahidol University, Bangkok, Thailand; ^6^Department of Genetics and Genome Biology, University of Leicester, Leicester, United Kingdom; ^7^Department of Infection Biology, Faculty of Infectious and Tropical Diseases, London School of Hygiene and Tropical Medicine, London, United Kingdom

**Keywords:** *Burkholderia pseudomallei*, *Burkholderia bacteriophage*, blood-induced prophage, hemoculture-isolated bacteriophage, melioidosis patient blood

## Abstract

Bacteriophages (phages), viruses that infect bacteria, are found in abundance not only in the environment but also in the human body. The use of phages for the diagnosis of melioidosis, a tropical infectious disease caused by *Burkholderia pseudomallei*, is emerging as a promising novel approach, but our understanding of conditions under which *Burkholderia* prophages can be induced remains limited. Here, we first demonstrated the isolation of *Burkholderia* phages from the hemocultures of melioidosis patients. The *B. pseudomallei*-positive hemoculture bottles were filtered to remove bacteria, and then phages were isolated and purified by spot and double agar overlay plaque assays. Forty blood samples (hemoculture-confirmed melioidosis) were tested, and phages were found in 30% of the samples. Transmission electron microscopy and genome analysis of the isolated phages, vB_HM387 and vB_HM795, showed that both phages are Myoviruses. These two phages were stable at a pH of 5–7 and temperatures of 25–37°C, suggesting their ability to survive in human blood. The genome sizes of vB_HM387 and vB_HM795 are 36.3 and 44.0 kb, respectively. A phylogenetic analysis indicated that vB_HM387 has homologs, but vB_HM795 is a novel Myovirus, suggesting the heterogeneity of *Burkholderia* phages in melioidosis patients. The key finding that *Burkholderia* phages could be isolated from the blood of melioidosis patients highlights the potential application of phage-based assays by detecting phages in blood as a pathogen-derived biomarker of infection.

## Introduction

Bacteriophages, or phages, are viruses of bacteria. They can be found abundantly in all natural environments, where they usually co-exist with their bacterial hosts. There are two life cycles of phages: the lytic and lysogenic cycles. During the lytic cycle, phages replicate, and progeny particles are released through lysis; such phage particles can be detected in the environment, including the human body. The presence of phages in the human body has been detected in various human microbiota studies (reviewed in Łusiak-Szelachowska et al., 2020), e.g., the skin (Hannigan et al., 2015), oral cavity (Pride et al., 2012), lungs (Shkoporov et al., 2022), gut (Minot et al., 2013), and urinary tract (Miller-Ensminger et al., 2018).

In contrast, during the lysogenic cycle, phages integrate their genomes into the bacterial chromosome and enter a dormant state. According to human microbiome studies, the majority of bacteria within the human body are lysogens harboring phage sequences within their genomes (Inglis et al., 2023). Importantly, these prophages can be induced under specific conditions, leading to bacterial host cell death and the release of newly assembled phages. Phages modify and regulate populations of bacterial commensals, playing a crucial role in the homeostasis of the human microbiota (Łusiak-Szelachowska et al., 2020). Prophage induction is typically triggered when bacterial DNA is damaged. The use of certain antibiotics, such as beta-lactamases or quinolones (Comeau et al., 2007), may activate the induction of prophages (Liu et al., 2022). Furthermore, under conditions of human infection by bacterial pathogens, neutrophils are the first cells of the immune system that attack the pathogens and secrete oxidative stress-inducing agents, which may trigger the production of prophages (Licznerska et al., 2016). Different phages that are specific to different human pathogens have been detected in a wide range of specimens, such as blood, serum, urine, and CSF (Blanco-Picazo et al., 2020; Shan et al., 2021).

There are several potential applications of phages in medicine, such as phage therapy, phage-based biocontrol, and phage-based diagnostics. The use of phages in assays for detecting bacteria was first reported over half a century ago, when an assay to detect *Salmonella* using the phage Felix 01 was described (Cherry et al., 1954). Currently, several phage-based diagnostic approaches to detect bacterial infections have been developed (Schofield et al., 2012), including phage replication assays for diagnosing pulmonary tuberculosis caused by *Mycobacterium tuberculosis* (McNerney et al., 2004), phage amplification assays to detect *Staphylococcus aureus* in blood cultures (Kingery et al., 2009), and quantitative real-time PCR (qPCR) monitoring of a reporter phage gene for the detection of *Yersinia pestis* (Sergueev et al., 2010).

Diagnostic approaches to target bacterial DNA or proteins are often adequate for culture-based diagnostics, but most such methods generally show low sensitivity and do not allow for the detection of low numbers of bacteria circulating in the blood. Remarkably, Shan et al. (2021) reported the development of a qPCR-based approach targeting the terminase large subunit (*ter*L) gene of *Borrelia* phage (Ter-qPCR) in blood samples as a means of detecting the presence of *Borrelia burgdorferi*. This approach provided higher sensitivity and demonstrated that PCR-based targeting of prophages may enable the detection of bacteria in blood samples (Shan et al., 2021). In *Burkholderia pseudomallei* diagnostics, phage-based approaches have not been extensively studied so far.

*Burkholderia pseudomallei*, an environmental bacterium that is widespread in soil and water in tropical regions worldwide, is highly prevalent across Southeast Asia and Northern Australia (Wiersinga et al., 2018). This bacterium is the causative agent of melioidosis, a neglected tropical infectious disease with a mortality rate of up to 40% in septicemia cases (Wiersinga et al., 2006). Infection can occur through inhalation or inoculation of the skin by contaminated soil or water (Currie et al., 2010). Although melioidosis is predominantly endemic in Southeast Asia and Northern Australia, recently, four non-travel-associated cases of melioidosis were reported in the United States, which were linked to an aromatherapy spray product imported from a melioidosis-endemic area (Gee et al., 2022). These cases highlight the risk of globally transmitted melioidosis disease from endemic areas. Currently, there is no vaccine available for melioidosis. Bacterial isolation from clinical specimens is considered the gold standard for melioidosis diagnostics (Leelarasamee and Bovornkitti, 1989). However, *B. pseudomallei* colonies are observed on an agar plate only after 2–4 days of incubation (Wikraiphat et al., 2015). Thus, the chances of making an early diagnosis are missed, and in severe melioidosis cases, patients die within 48 h after hospitalization (Kung et al., 2011). Therefore, rapid methods for the diagnosis of melioidosis are needed.

Genome analysis of prophages on *B. pseudomallei* genomes found the majority of *B. pseudomallei* strains are commonly lysogenized by one, two, or three prophages, which are highly conserved in their sequences and genomic organization (Ronning et al., 2010). Moreover, the survey of divergent putative genomic islands and clinically significant *B. pseudomallei* populations has demonstrated an important role of prophage-associated genes for the genetic diversity of this pathogen or may lead to altered phenotypes such as colony morphology (DeShazer et al., 2019). Although several studies have demonstrated the presence of prophages sequences in the genome of *B. pseudomallei* isolated from melioidosis patients and *in vitro* studies have revealed that prophages could be induced from the bacteria (Kvitko et al., 2012; Hammerl et al., 2020), there are no reports on the detection and characterization of free phages from the blood of melioidosis patients infected with *B. pseudomallei*. We hypothesize that *B. pseudomallei* prophages can be induced *in vivo*, and phage particles released into the blood stream of melioidosis patients could be detected and used as a biomarker for *B. pseudomallei* infection. This hypothesis is based on the consideration that no *B. pseudomallei*-specific phages should be present in the blood of non-infected individuals.

In this study, we report the development of an assay for the isolation of *B. pseudomallei*-specific phages from hemocultures. To the best of our knowledge, this is the first report on the isolation of *B. pseudomallei*-specific phages from melioidosis patients’ hemoculture. The isolated *Burkholderia* phages were characterized, and their biological and genomic properties were assessed. Whole-genome sequence analysis of two of the isolated phages revealed that the *Burkhoderia* phages were distinct. The positive isolation of *Burkholderia* phages from melioidosis patients suggests that developing an assay to detect free phages in clinical samples instead of targeting bacterial pathogens may be an alternative approach to improve the diagnosis of melioidosis.

## Materials and methods

### Ethics statement

Leftover hemoculture samples that were culture-positive for *B. pseudomallei* were taken from Srinagarind Hospital, Faculty of Medicine, Khon Kaen University. Retrievals of patients’ hemoculture samples were reviewed and approved by the Khon Kaen University Ethics Committee for Human Research based on the Declaration of Helsinki and the ICH Good Clinical Practice Guidelines (approval number: HE651180). In addition, this research project was approved by the Institutional Biosafety Committee (IBC), Faculty of Medicine, Siriraj Hospital, and Faculty of Tropical Medicine, Mahidol University. The approval certificate numbers are SI 2022–013 and MU 2023–002.

### Bacterial strains

Bacterial strains used in this study are listed in Table 1. *B. thailandensis* strain E174 was chosen as a propagation strain for phage isolation and purification. Bacteria were cultured on Luria–Bertani (LB) agar (Hardy Diagnostics, United States) and incubated at 37°C for 18–24 h. To obtain mid-log phase bacteria, 30 μL of overnight-cultured bacteria was sub-cultured into 3 mL of LB broth (Thermo Fisher Scientific, United States) and incubated at 37°C for approximately 4 h.

**Table 1:** List of bacteria used in this study.

Bacterial species	Sources	Total strains	Strains	Ref.
*B. pseudomallei*	Soil, Thailand	5	SBPTHE0008, SBPTHE0412, SBPTHE0024, SBPTHE0031, SBPTHE0358	(1)
	Clinical, Thailand	6	K96243, H576a, H3073a, H1710a, H956a, H239a	(1)
	Soil, Australia	3	SBPAU016a, SBPAU017a, SBPAU018	(2)
	Water, Australia	1	SBPAU491	
	Clinical, Australia	4	HBPAU015, HBPAU019, HBPAU024a, HBPAU026	(2)
*B. thailandensis*	Thailand	41	DW503, DV1, D1, E152, E153, E154, E158, E159, E169, E173, E174, E175, E177, E184, E188, E192, E201, E202, E205, E207, E228, E234, E246, E253, E264, E27, E274, E332, E352, E354, E360, E421, E426, E427, E430, E433, E435, E436, E438, E440, E441	(1)
*B. thailandensis* capsule variant (BTCV)	Thailand	20	E555, SBXCB001, SBXCB002, SBXCC001, SBXCC003, SBXCC004, SBXPL001, SBXPL002, SBXPL003, SBXPL015, SBXPR001, SBXPR002, SBXPR005, SBXRY003, SBXRY005, SBXSR001, SBXSR003, SBXSR004, SBXSR007, WBXUBA33005104	(1)
*B. oklahomensis*	Human, USA	1	NCTC 13387	
*B. multivorans*	Human, UK	1	LMG16660	
*B. vietnamensis*	Human, Vietnam	1	LMG6999	
*B. cepacia*	Human, Thailand	4	NVDK30, NVDK31, BC10744, BC44	
*A. baumannii*	Human, Thailand	3	AB131, 90,855, AB153	
*Salmonella* Enteritidis	Human, Thailand	3	No.8, No. 37, No. 219	
*E. coli*	Human, Thailand	1	No.1	

Mahidol-Oxford Tropical Medicine Research Unit (MORU). Professor Bart Currie, Menzies School of Health Research, Charles Darwin University, Darwin, Australia.

### Hemoculture and phage isolation

Hemoculture bottles that were positive for *B. pseudomallei* were collected and kept at 4°C until phage isolation. After centrifugation at 8,000× *g* for 10 min, 10 mL of the hemocultures was aspirated and filtered through a 0.45-μm membrane filter (Whatman, United Kingdom) to remove cell debris. Then, the filtrated samples were stored at 4°C until phage screening by spot assay, following the method previously described by Clokie and Kropinski (2009) with some modifications. Essentially, 10 μL of the filtrate hemoculture samples were spotted onto the LB top agar (0.35%) containing *B. thailandensis* strain E174 before incubation at 37°C for 16–18 h. The next day, a single clear plaque was picked by using a sterile end-cut-yellow pipette tip. Then, the pipette tip was put into a microcentrifuge tube containing 1 mL of SM buffer (100 mM NaCl, 8 mM MgSO_4_·7H_2_O, 50 mM Tris–HCl, pH 7.5) and swirled to release the piece of agar into the buffer. The collected sample was maintained at 4°C until the next step of purification by double agar overlay plaque assay.

### Phage purification

A single clear zone collected from spot assays was purified by double agar overlay plaque assay (Clokie and Kropinski, 2009) for at least 10 rounds. Briefly, a mixture of 100 μL of mid-log bacterial culture, 100 μL of the filtered sample, and 5 mL of LB top agar (0.35% agar) was poured on to an LB bottom agar (1% agar) and incubated overnight at 37°C. Phage propagation was performed by double agar overlay plaque assay with approximately 30 plates per sample. SM buffer was added to each plate before plate lysates were collected, centrifuged, and filtered through a 0.45-μm filter membrane. To concentrate, the filtrated lysate was mixed with 10% (w/v) PEG8000 (Sigma-Aldrich, Inc., United States) and 0.1 M NaCl (Sigma-Aldrich, Inc., United States). After centrifugation, the pellet was dissolved in SM buffer and collected at 4°C for further characterization.

### Phage morphology by transmission electron microscopy

Phages were negatively stained and observed morphology under transmission electron microscopy (TEM), according to the previously described study (Imam et al., 2019). Briefly, phages (10^8^ particles/mL) were negatively stained with 2% uranyl acetate on a Formvar–carbon-coated copper grid (200 mesh). After staining, each sample was imaged using the JEM 1400 transmission electron microscope (JEOL Co., Japan) with an accelerating voltage of 80 kV. Images of randomly selected phage particles (typically, 8–12 per sample) were then analyzed using ImageJ (version 1.49) software to estimate the approximate sizes of the phage capsid and tail.

### Determination of the phage host range

The host range of the isolated phages was determined using spot assays. Ten microliters of high-titer phages were spotted on various bacterial strains, including 18 strains of *B. pseudomallei*, 41 strains of non-encapsulated *B. thailandensis*, 20 strains of encapsulated *B. thailandensis* (*B. thailandensis* capsule variant; BTCV), 1 strain of *B. oklahomensis*, 1 strain of *B. vietnamensis*, 1 strain of *B. multivorans*, 4 strains of *B. cepacia*, and other Gram-negative bacteria, including 3 strains of *Acinetobacter baumannii*, 3 strains of *Salmonella enterica*, and 1 strain of *E. coli* (Table 1). Independent experiments were repeated three times.

### Thermal and pH stability of phages

For thermal stability determination, phage suspension was incubated at 25°C, 37°C, 45°C, and 55°C, respectively. For pH stability determination, phages were mixed with SM buffer at a pH range of 2–11. After incubation for 30 min, 1, 3, 5, and 24 h, the thermal and pH-treated phage samples were taken for 10-fold serial dilution with SM buffer before phage titers were determined by spot assay. Independent experiments were repeated three times.

### Phage genomic DNA extraction

Phage genomic DNA (gDNA) was extracted by the phenol–chloroform–isoamyl alcohol (PCI) extraction protocol (Sambrook and Russel, 2001). Briefly, phage suspension (10^8^ PFU/mL) was mixed with 1 M MgCl_2_ (Sigma-Aldrich, Inc., United States), DNAseI, and RNAseA (Thermo Fisher Scientific, United States) before incubation for 30 min at room temperature. Then, Proteinase K (Thermo Fisher Scientific, United States), 0.5 M EDTA, and 10% SDS were added. After incubation at 55°C for 1 h, the mixture was treated with phenol:chloroform:isoamyl alcohol (25:24:1) and then centrifuged. Only the top aqueous layer was collected and mixed with chilled, equal amounts of 95% ethanol and 3 M sodium acetate (Sigma-Aldrich, Inc., United States). The mixture was then incubated overnight at −20°C before centrifugation, and the pellets were washed with 70% ethanol before dissolving the dried pellets in nuclease-free water and stored at −20°C or − 80°C for long-term storage.

### Phage whole-genome sequencing, assembly and annotation

Genomic libraries of phages were prepared using the Illumina DNA Library Prep kit (Illumina, Inc., United States) before sequencing using an Illumina MiSeq. Low-quality bases were trimmed using sickle v1.33 and Trimmomatic v0.39 (Joshi and Fass, 2011). Then, the trimmed FASTQ files were assembled using SPAdes v.3.6.0 (Bankevich et al., 2012) and Unicycler v0.4.8 (Wick et al., 2017). Phage genomes were reordered and oriented by CONTIGuator (Galardini et al., 2011) and manually using the Geneious program. Sequences were identified as ORFs and annotated by Prokka v1.12 (Seemann, 2014) using a custom “Caudovirales” database. Putative functions were assigned and searched against BLASTP^1^ and Pfam (Mistry et al., 2021). Phage genome maps were then generated and visualized using Artemis version 17.0.1 (Rutherford et al., 2000).

### Phage genome comparisons

For comparative genomics, average nucleotide identity (ANI) was calculated using FastANI v1.3 (Jain et al., 2018). To determine phylogenetic relationships among these viruses, phage whole genomes were aligned using Multiple Alignment Fast Fourier Transform (MAFFT) v7.505 (mafft --auto input > output) to build nucleotide alignments based on the aligned amino acid sequence (Katoh et al., 2002). Additionally, phage genome sequences were uploaded into ViPTree (Nishimura et al., 2017) to analyze phylogenetic phage proteomes. The evolutionary history was inferred by using the neighbor-joining method (Saitou and Nei, 1987). Then, the phylogenetic trees were visualized using the Interactive Tree of Life v6.5.8 (iTOL) programs (Letunic and Bork, 2021). Comparative genome contents were visualized and compared using Clinker v0.0.25 (Gilchrist and Chooi, 2021).

### Statistical analysis

Statistical analysis was performed using GraphPad Prism 8.0 software (GraphPad, CA, United States). The one-way ANOVA was applied to compare the variance in the group means. *p*-values less than 0.05 were considered statistically significant (*p < 0.05, **p < 0.01, and ***p < 0.001, ns; not significant).

## Results

### The presence of *Burkholderia pseudomallei*-specific phages in melioidosis-positive hemoculture

To see if phages could be isolated from melioidosis patients, 40 hemoculture-positive cultures of *B. pseudomallei* were obtained from the Srinagarind Hospital, Faculty of Medicine, Khon Kaen University. The cultures were filtered, and the filtrates were spotted on the lawns of 41 *B. thailandensis* strains (Table 1). Initially, optimization showed that *B. thailandensis* strains E174 and E332 were sensitive to hemoculture-isolated phages; these two strains were then selected as phage isolation strains. Interestingly, 12 samples from 40 hemoculture-positive cultures were positive for phages on these strains, as indicated by a spot assay on a lawn of *B. thailandensis* (30.0% isolation rate). These results were then confirmed by the double agar overlay plaque assay. Several phage plaque morphologies were observed on bacterial lawns.

**Figure 1:**
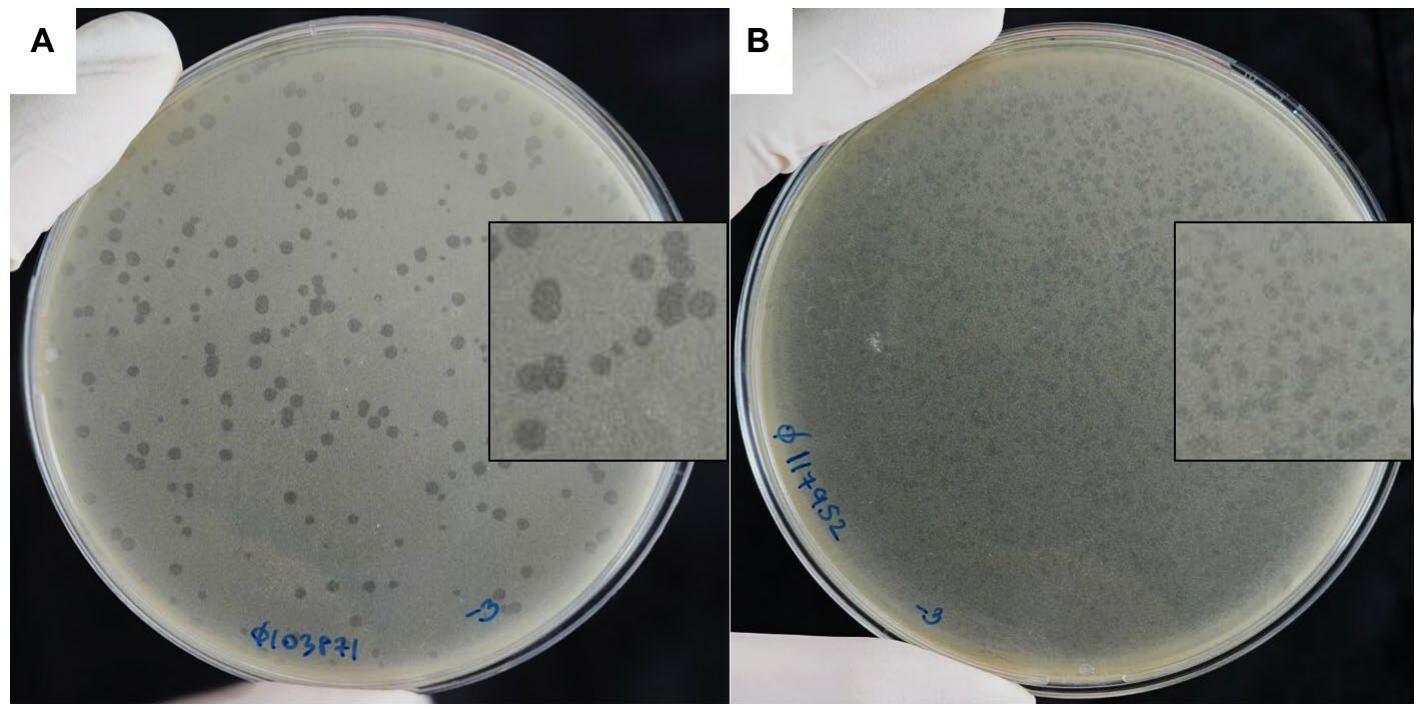
Plaque morphologies of the hemoculture-isolated *Burkholderia* phages. *Burkholderia* phages vB_HM387 **(A)** and vB_HM795 **(B)** were isolated from different hemoculture bottles of melioidosis patients using *B. thailandensis* strain E174 as a propagation host.

Two of the phages that produced plaques with distinct morphologies were selected for further study and named vB_HM387 and vB_HM795. After several rounds of single plaque purification, vB_HM387 formed small turbid plaques (approximately 1–3 mm in diameter) with rough edges and a clear spot at the center (bull eyes’ morphologies), whereas vB_HM795 formed small turbid plaques (approximately 1–2 mm in diameter) with no edges on *B. thailandensis* strain E174 lawn (Figure 1). This is the first report showing that phages can be isolated from hemoculture-positive cultures from melioidosis patients and that these phages can have distinct plaque morphologies on *B. thailandensis* lawns.

To investigate if human blood triggers prophage release from bacterial genomes, *B. thailandensis* was spiked into fresh normal human venous blood and PBS, followed by assessing viable bacteria and free phages. We found that free phages were detected in *B. thailandensis*-spiked human venous blood, but no free phages were detected in *B. thailandensis*-spiked PBS (Supplementary material). Additionally, after incubation, no viable *B. thailandensis* was detected in *B. thailandensis*-spiked human venous blood, but *B. thailandensis* remained viable in PBS. These findings imply that *Burkholderia* prophages could be induced by interactions of bacteria with human blood.

### Morphological characteristics of the isolated phages

**Figure 2:**
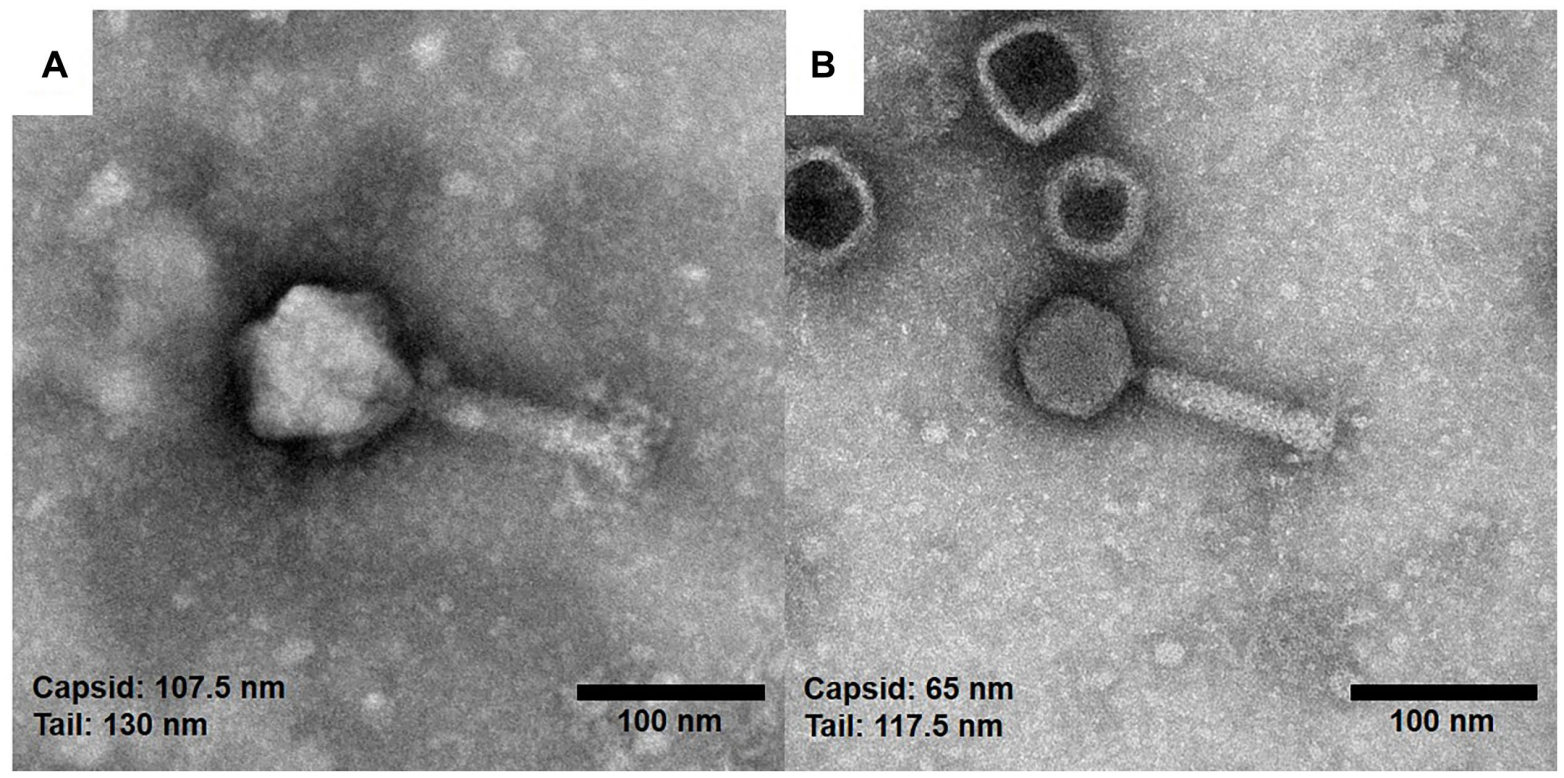
Electron micrographs of the hemoculture-isolated *Burkholderia* phages. The purified *Burkholderia* phages vB_HM387 **(A)** and vB_HM795 **(B)** are Myoviruses characterized by the possession of hexagonal capsids (107.5 and 65.0 nm) wide with long contractile tails, and the tails were approximately 130.0 and 117.5 nm long, respectively.

To observe the morphology of the hemoculture-isolated phages, TEM analysis was undertaken. Based on the TEM assessment of phage tail morphologies, two selected phages (vB_HM387 and vB_HM795) are Myoviruses in the order Caudovirales (Figure 2). These phages are characterized by the presence of hexagonal capsids (approximately 102.6–107.5 and 63.6–68.2 nm width) and long contractile tails (approximately 120.8–130.0 nm and 109.1–117.5 nm in length) for vB_HM387 and vB_HM795, respectively.

### Lysis spectrum of the isolated phages

The lytic activities of vB_HM387 and vB_HM795 phages on different strains of *Burkholderia* species and other related Gram-negative bacteria were assessed using spot assays. The results revealed that phages vB_HM387 and vB_HM795 could lyse 6/19 and 11/19 isolates of *B. pseudomallei* and 35/61 and 53/61 isolates of *B. thailandensis*, respectively (Table 2). Among six clinical *B. pseudomallei* strains isolated from Thailand, each of the phages vB_HM387 and vB_HM795 could lyse 2/6 different *B. pseudomallei* strains and shared the ability to lyse the other 2/6 different strains of *B. pseudomallei*. Thus, there were a total of four *B. pseudomallei* strains that could be lysed by each phage. Differently, among five soil *B. pseudomallei* strains, phage vB_HM795 could lyse 4/5 strains, whereas phage vB_HM387 could lyse 2/5 strains, with two strains overlapping (Supplementary Table S2). Among 61 *B. thailandensis* isolates composed of non-capsulated and capsulated *B. thailandensis* or *B. thailandensis* that express a *B. pseudomallei*-like capsule (BTCV), the infection rates against non-capsulated *B. thailandensis* were 80.49% (33/41) and 82.93% (34/41) for vB_HM387 and vB_HM795, respectively. For the ability of hemoculture-isolated phages to infect BTCV (Sim et al., 2010), we found that vB_HM387 could infect only two out of 20 tested BTCV strains, whereas strikingly, vB_HM795 could infect 19 of 20 BTCV strains. These results indicated that phages vB_HM387 and vB_HM795 had a broad spectrum to infect *B. thailandensis*, but phage vB_HM795 had a wider range to infect BTCV strains than vB_HM387. Neither phage could infect other related species of *Burkholderia* or Gram-negative bacterial strains (Table 2), suggesting the specificity of two isolated phages against *B. pseudomallei* and *B. thailandensis*.

**Table 2:** Lytic activity of the hemoculture-isolated *Burkholderia* phages vB_HM387 and vB_HM795 against *Burkholderia* spp. and other Gram-negative bacteria.

Bacterial species	Sources	Total strains	vB_HM387 number of lysis (%)	vB_HM795 number of lysis (%)
*B. pseudomallei*	Soil, Thailand	5	2 (40.00)	4 (80.00)
	Clinical, Thailand	6	4 (66.67)	4 (66.67)
	Soil, Australia	3	0 (0.00)	1 (33.33)
	Water, Australia	1	0 (0.00)	0 (0.00)
	Clinical, Australia	4	0 (0.00)	2 (50.00)
*B. thailandensis*	Thailand	41	33 (80.49)	34 (82.93)
BTCV	Thailand	20	2 (10.00)	19 (95.00)
*B. oklahomensis*		1	0 (0.00)	0 (0.00)
*B. multivorans*		1	0 (0.00)	0 (0.00)
*B. vietnamensis*		1	0 (0.00)	0 (0.00)
*B. cepacia*		4	0 (0.00)	0 (0.00)
*A. baumannii*		3	0 (0.00)	0 (0.00)
*Salmonella* spp.		3	0 (0.00)	0 (0.00)
*E. coli*		1	0 (0.00)	0 (0.00)

Importantly, vB_HM387 and vB_HM795 could infect both clinical and environmental *B. pseudomallei* strains isolated from Thailand. Interestingly, one hemoculture-isolated phage (vB_HM795) from a melioidosis patient from Thailand could infect and cause lysis of a *B. pseudomallei* strain isolated from Australia (Table 2). Taken together, there are differences in biological properties between vB_HM387 and vB_HM795, in which vB_HM795 showed a broader spectrum than vB_HM387 to infect BTCV and Australian *B. pseudomallei* strains.

### Phage resistance to human blood stresses

**Figure 3:**
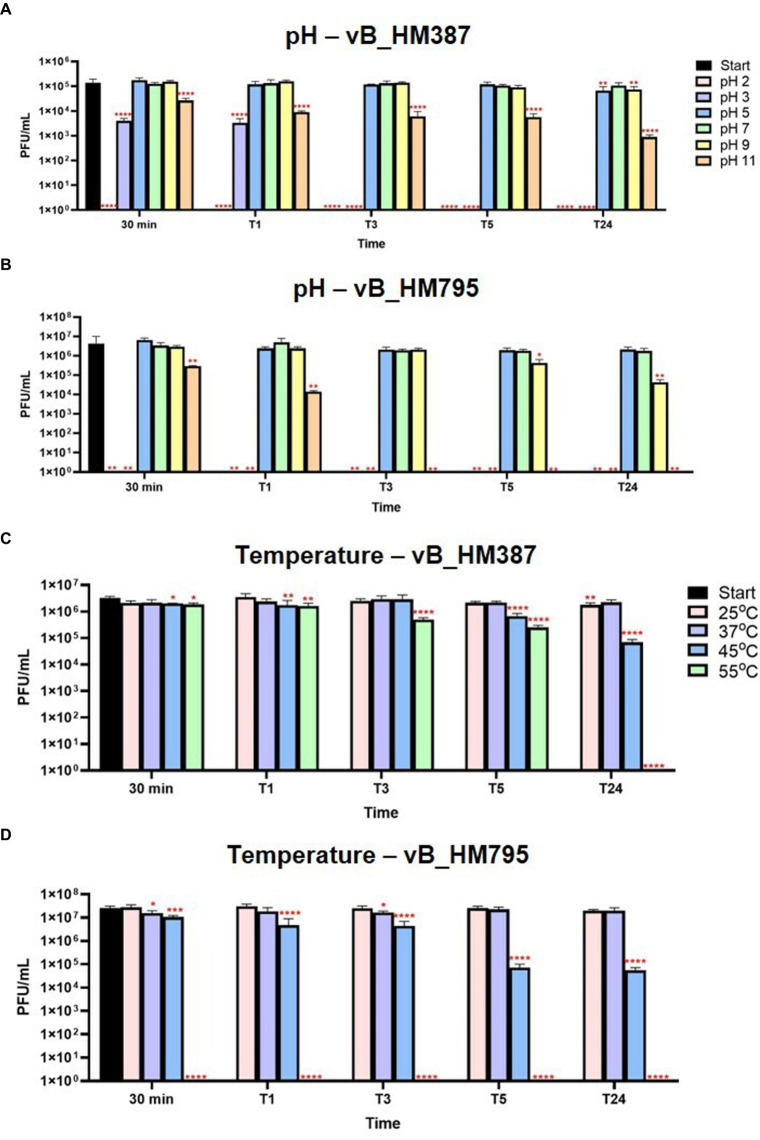
Thermal and pH stabilities of phages vB_HM387 and vB_HM795. Phages vB_HM387 **(A,C)** and vB_HM795 **(B,D)** were either exposed to varying pH ranging from 2 to 11 **(A,B)** or incubated at varying temperatures **(C,D)** for 30 min, 1, 3, 5, and 24 h before being assessed by spot assays. Significances were determined using one-way ANOVA with Dunnett’s multiple comparisons *post-hoc* test across all samples. The data demonstrated the mean ± standard deviation (SD) of triplicate experiments. The asterisk indicates statistical significance (*p < 0.05, **p < 0.01, ***p < 0.005, and ****p < 0.001).

Assessing the effects of pH and temperature on the stability of phage virions may provide an indication of how phages can withstand the stress imposed by human blood. To determine pH stability, phages were exposed to varying pH ranges from 2 to 11 before being assessed for viability by spot assays. The results showed that phage vB_HM387 was inactivated at very acidic conditions (pH 2–3) but was stable at pH 5–9. Additionally, the viability of vB_HM387 was only slightly reduced following incubation at the strong alkaline (pH 11) condition for 24 h (T24; Figure 3A). Whereas, phage vB_HM795 was sensitive to strongly acidic (pH 2–3) and strong alkaline (pH 11), but it was stable at pH 5–7, and its viability only slightly reduced at 5-h post-treatment, and a further reduction in viability was observed after 24-h incubation at pH 9 (Figure 3B). In conclusion, the major difference between these two phages is that vB_HM387 was more resistant to alkaline conditions than vB_HM795.

To determine the thermal tolerance of the phages, they were incubated at varying temperatures (25, 37, 45, and 55°C) before assessing their viability by spot assay. As shown in Figures 3C,D, both phages vB_HM387 and vB_HM795 were stable at 25 and 37°C. However, the titers of these two phages were significantly reduced after exposure to 45°C. At the higher temperature (55°C), phage vB_HM387 remained viable until T5 but was completely inactivated by T24, whereas phage vB_HM795 could not tolerate 55°C, even for a short period of incubation. In conclusion, these two phages showed different thermal stability, as phage vB_HM387 showed more tolerance at high temperatures than vB_HM795.

### Whole-genome analysis

To investigate the genetic contents of the isolated phages vB_HM387 and vB_HM795, their genomes were sequenced and analyzed (deposited in GenBank accession numbers: OR990504 and OR990505, respectively). Analyses of the genomes of phages vB_HM387 and vB_HM795 indicated that they consist of double-stranded DNA genomes of 36.3 and 44.0 kb in size and 65.3 and 63.5% GC contents, respectively. No tRNAs were detected in either of the phages, and 43 and 51 putative open reading frames (ORFs) were predicted in the genomes of vB_HM387 and vB_HM795, respectively.

**Figure 4:**
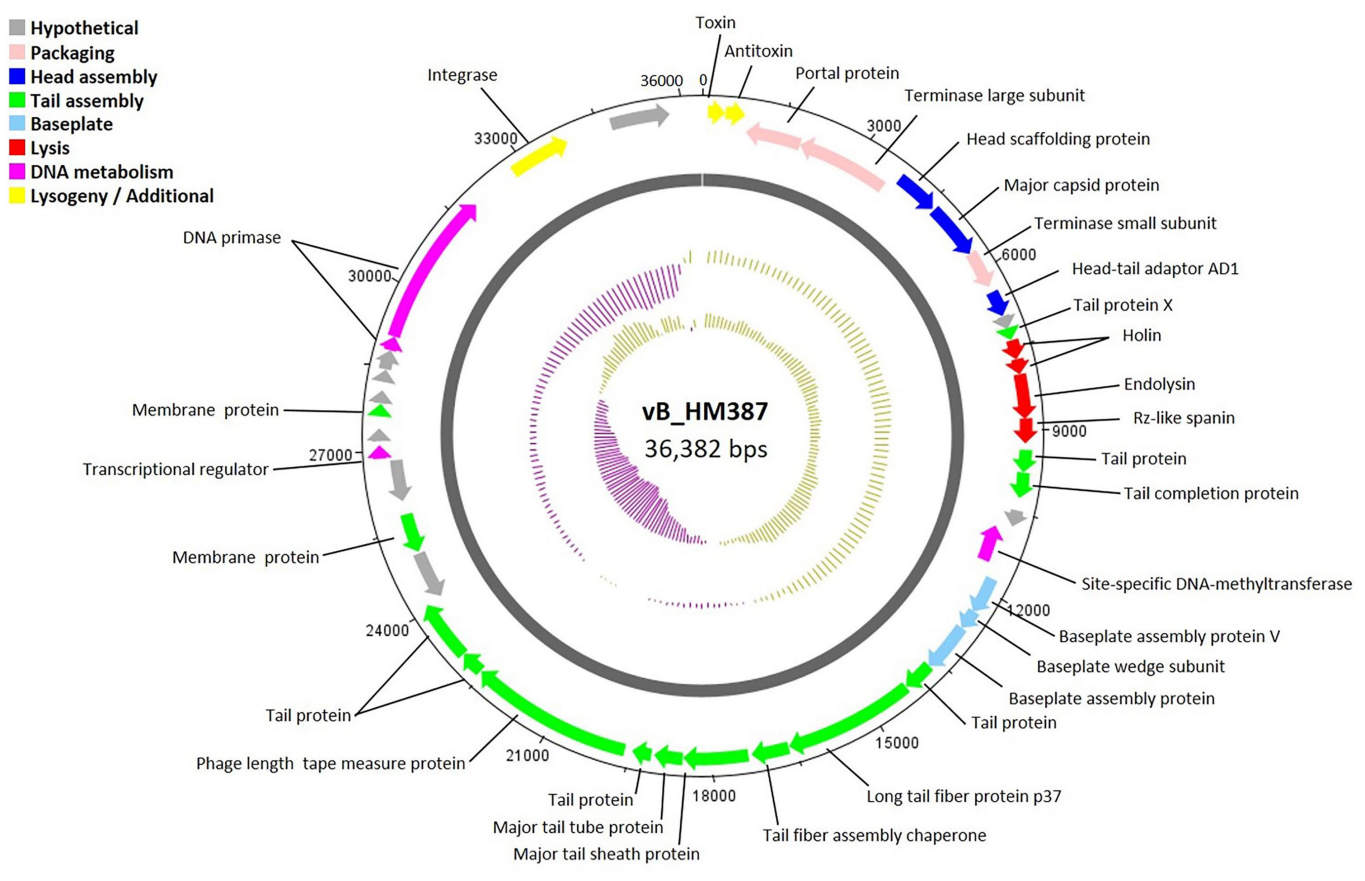
Circular genome map of *Burkholderia* phage vB_HM387. Phage genome visualization performed using the DNAPlotter is shown by the dark gray circle. Genomes are organized into eight functional modules, represented by the colored bars and indicated in the genes’ annotation list. The outside circle shows the scale in bases, with 0 representing the origin of replication. The inner circle represents the value of the GC skew: yellow for positive and purple for negative. The outer circle represents the value of the GC plot: purple for G + C content below the average level and yellow for G + C content above the average level.

The circular genome map of phage vB_HM387 is shown in Figure 4, with 9 of 43 genes (20.93%) encoding hypothetical proteins. The remaining 34 ORFs were functionally annotated, as predicted by RAST and BLASTP. Genome analysis revealed five major functional groups of genes/proteins: a packaging module, a phage structure module, a lysis module, a DNA metabolism module, and an additional module. The structural module comprised 20 genes encoding proteins required for host recognition and structural assembly: tail-associated proteins, baseplate-associated proteins, and head-associated proteins. Phage vB_HM387 encodes a major tail sheath (gp25) and major tail tube proteins (gp26), which are normally found in Myoviruses. Predicted morphology was confirmed by observation under transmission electron microscopy (Figure 2A). The lysis gene module comprised four genes required for host cell lysis (two holins, the N-acetylmuramidase family protein, and LysB). A gene encoding integrase, which is required for lysogeny, was identified, indicating that phage vB_HM387 was an obligate lysogenic phage. Interestingly, the type II toxin–antitoxin (TA) addition module genes were found in this phage genome.

**Figure 5:**
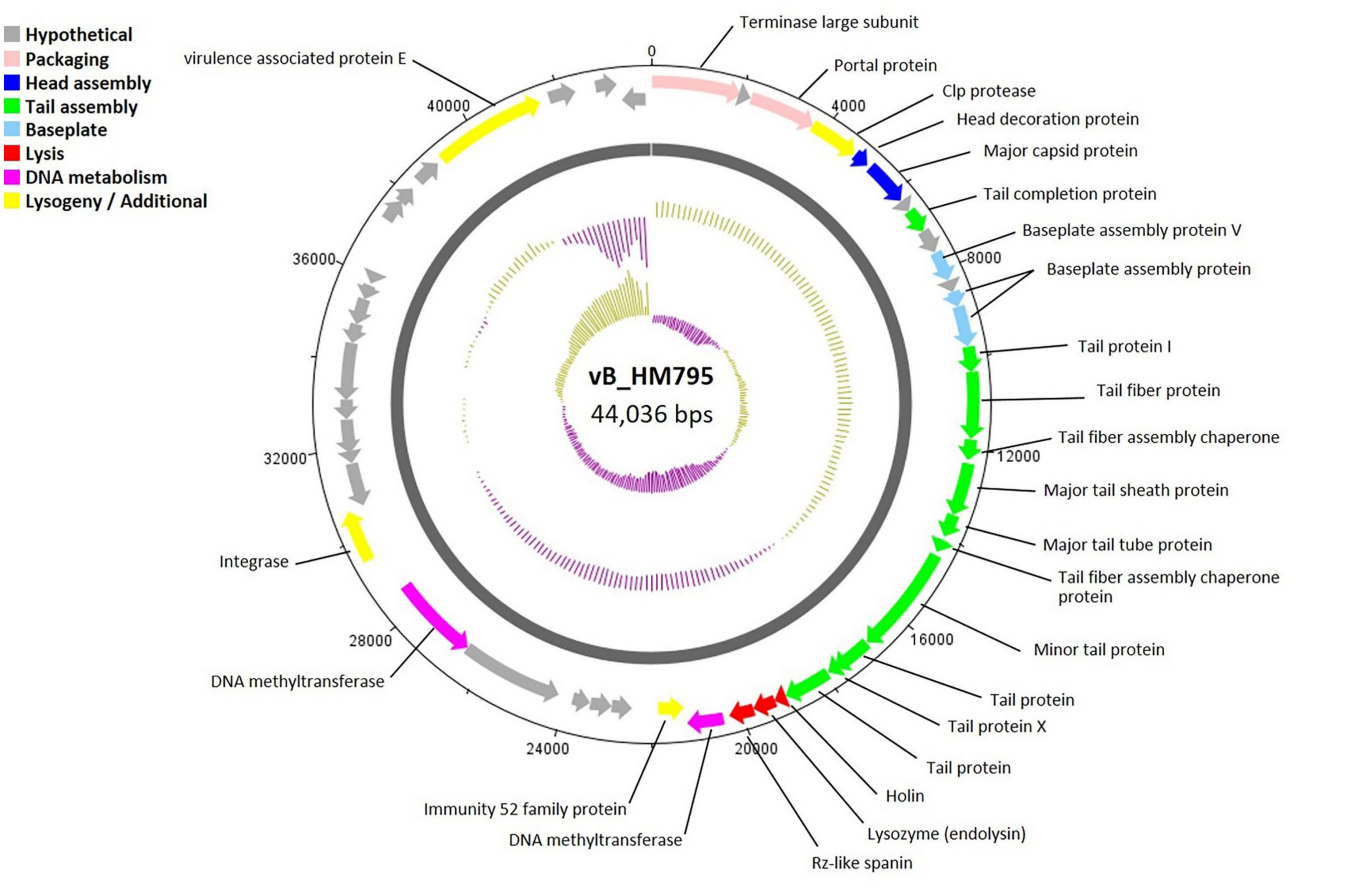
Circular genome map of *Burkholderia* phage vB_HM795. Phage genome visualization performed using the DNAPlotter is shown by the dark gray circle. Genomes are organized into eight functional modules, represented by the colored bars and indicated in the genes’ annotation list. The outside circle shows the scale in bases, with 0 representing the origin of replication. The inner circle represents the value of the GC skew: yellow for positive and purple for negative. The outer circle represents the value of the GC plot: purple for G + C content below the average level and yellow for G + C content above the average level.

A circular genome map of phage vB_HM795 is shown in Figure 5, with 23 of 50 genes (46.00%) encoding hypothetical proteins. The products of 27 ORFs were functionally annotated, and the genome analysis revealed five major functional groups as described above. The structural module comprised 16 genes. Phage vB_HM795 also encodes a major tail sheath (gp17) and major tail tube proteins (gp18), suggesting that these phages are Myoviruses (Figure 2B). The lysis gene module comprised three genes required for host cell lysis (holin, lysozyme, and spanin). A gene encoding integrase was also identified, indicating that this phage is a temperate phage. Interestingly, genomic analysis of the phage vB_HM795 revealed a gene encoding a known virulence-associated protein E (*vapE*) of *B. pseudomallei* (CAJ2861095). This finding is correlated with a previous report that prophages frequently harbor gene-encoding virulence-associated proteins (*vap*), which play roles in bacterial pathogenesis (Canchaya et al., 2003).

### Comparative genome analysis of isolated phages and other *Burkholderia* phages

**Figure 6:**
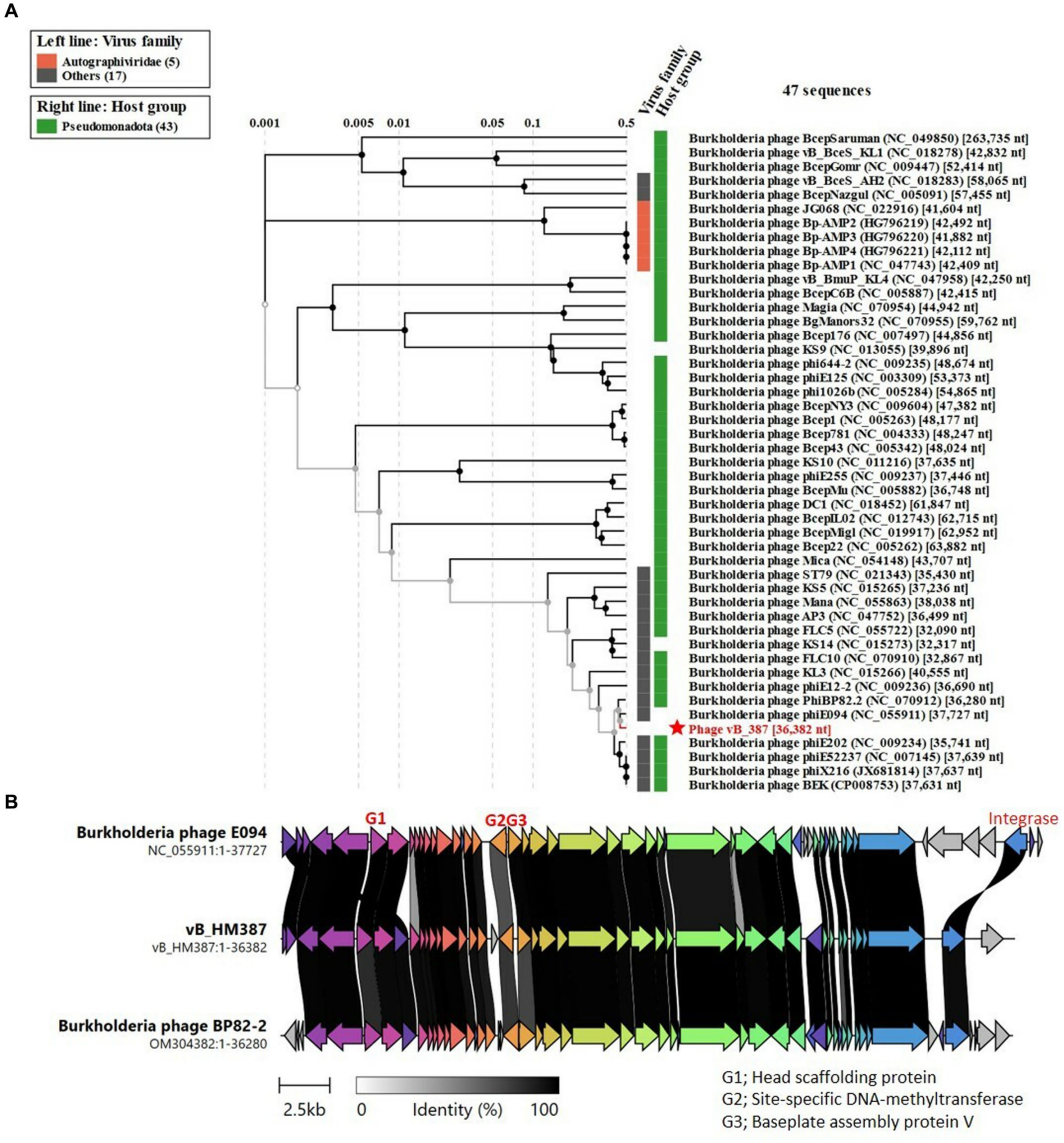
Comparative genome analysis of phage vB_HM387 to other *Burkholderia* phages in the NCBI database. The whole-genome sequence of phage vB_HM387 was uploaded into ViPTree (Nishimura et al., 2017) to analyze phylogenetic phage proteomes **(A)**. The tree includes 47 related *Burkholderia* phages, with the phage vB_HM387 in this study highlighted with a red star. Taxa are also annotated with the virus family (*Autographiviridae* and others) and the bacterial host (*Pseudomonadota*). Similarities in the genome alignment of phage vB_HM387 and *B. pseudomallei* phages phiE094 and phiBP82.2 were compared by Clinker **(B)**. Four genome modules were compared, including the head assembly module, tail assembly module, lysis (L) module, and accessory modules (lysogeny/DNA metabolisms). The arrow’s colors represent the gene clusters, which encode similar proteins. Gray bars shaded represent the percentage of amino acid identity from 1 to 100%.

In order to study the phylogenetic relationships between vB_HM387, vB_HM795, and other phages, whole-genome sequences of phages vB_HM387 and vB_HM795 were uploaded into ViPTree to construct phylogenetic trees based on the whole-genome encoded amino acid sequences. Figure 6A shows the phylogenetic tree of phage vB_HM387. This revealed that vB_HM387 belongs to the same species as seven other *Burkholderia* phages in the NCBI database, including having 95.05% nucleotide identity to phage phiE094 (90% coverage, NC_055911.1), 98.93% nucleotide identity to phiBP82.2 (89% coverage, NC_070912.1), 98.29% nucleotide identity to phiE202 (86% coverage, NC_009234.1), 96.33% nucleotide identity to phiE52237 (80% coverage, NC_007145.2), 96.25% nucleotide identity to phiBEK (80% coverage, CP008753.1), and 96.14% nucleotide identity to phiX216 (80% coverage, JX681814.1). To confirm these findings, the most similar phages were selected for a comparative genomic synteny and similarity analysis using Clinker (Figure 6B). Phage vB_HM387 shows a high similarity to phages E094 and BP82.2, with some sequence variability in genes encoding phage capsid scaffolding protein, site-specific DNA-methyltransferase, and baseplate assembly proteins.

Moreover, the vB_HM387 phage is also conserved in the bioinformatically identified prophage-like elements GI15 of *B. pseudomallei* K96243 and PI-E264-2 of *B. thailandensis* E264 (Ronning et al., 2010). Searching the NCBI database using the BLAST algorithm revealed that phage vB_HM387 shows high identity to various *B. pseudomallei* strains (93% coverage, >98% identity) isolated from various regions, including Thailand, e.g., strain 1026b (CP002833.1), Malaysia, e.g., UKMH10 (LR595892.1), Japan, e.g., strain Tokushima (AP028081.1), and Australia strains, e.g., MSHR5864 (CP017048.1), and MSHR6755 (CP017046.1). These results indicated that the phage vB_HM387 can exist as a prophage in many strains of *B. pseudomallei* isolated from multiple countries.

**Figure 7:**
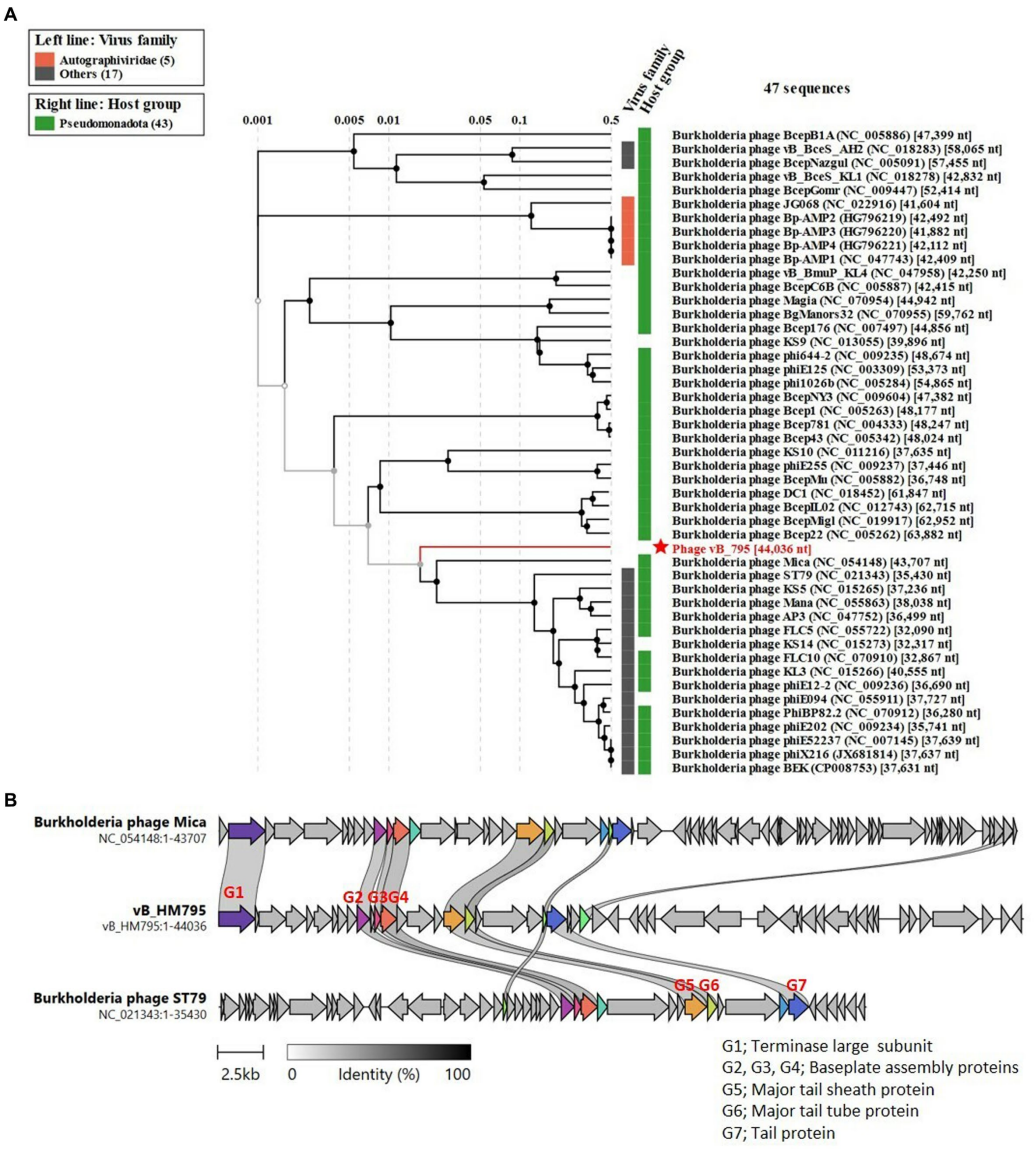
Comparative genome analysis of phage vB_HM795 to other *Burkholderia* phages in the NCBI database. The whole-genome sequence of phage vB_HM795 was uploaded into ViPTree (Nishimura et al., 2017) to analyze phylogenetic phage proteomes **(A)**. The tree includes 47 related *Burkholderia* phages, with the phage vB_HM795 in this study highlighted with a red star. Taxa are also annotated with the virus family (*Autographiviridae* and others) and the bacterial host (*Pseudomonadota*). Similarities in the genome alignment of phage vB_HM795, *B. pseudomallei* phages, Mica and ST79, were compared by Clinker **(B)**. Four genome modules were compared, including the head assembly module, tail assembly module, lysis (L) module, and accessory modules (lysogeny/DNA metabolisms). The arrow’s colors represent the gene clusters, which encode similar proteins. Gray bars shaded represent the percentage of amino acid identity from 1 to 100%.

Figure 7A shows the phylogenetic tree of phage vB_HM795. Our analysis revealed that phage vB_HM795 represents a unique cluster, far from the other previous *Burkholderia* phages in the NCBI database. An NCBI BLASTN search revealed that phage vB_HM795 shared only 3% coverage with other phages. To confirm this, the most similar *Burkholderia* phages (Mica and ST79) were selected, and a comparative genomic similarity was carried out using Clinker (Figure 7B). Similar to the phylogenetic tree analysis, vB_HM795 shows less homology to other *Burkholderia* phages, including vB_HM387, indicating that phage vB_HM795 can be considered a novel phage. Phage vB_HM795 has unique DNA packaging, head and tail biosynthesis, host lysis, lysogeny, or DNA replication genes. However, some genes show similar to but low percentage identity to the most similar *Burkholderia* phages, Mica and ST79, including large terminase, baseplate assembly protein V, baseplate assembly protein, baseplate J/gp47 family protein, major tail sheath protein, and contractile injection system protein.

Searching the NCBI database using the BLAST algorithm revealed that phage vB_HM795 shows a high identity to a fragment of the bacterial chromosome of *B. pseudomallei* strain HNBP001 isolated from China (CP038805.1) with 97% coverage and 98.01% identity. Additionally, phage vB_HM795 also showed some similarity to other *B. pseudomallei* strains (>83% coverage and > 98%), such as the Chinese isolated strain 14 M0960418 (CP019042.1), the Japan-isolated strain GTC3P0054 (AP028075.1), the Singapore isolated strain AW17-22 (CP073737.1), the Ecuador isolated strain 7,894 (CP009535.1), and the India isolated strain VBP399 (CP071757.1). Searching vB_HM387 and vB_HM795 against the *Burkholderia* group (taxid:119060) deposited in the GenBank database with a maximum number of 5,000 target sequences revealed that phages vB_HM387 and vB_HM795 showed high sequence similarity (>60% coverage, >90% identity) to 98 genomes of *B. pseudomallei* strains. Specifically, vB_HM387-like and vB_HM795-like prophages were found in 40/98 (40.82%) and 30/98 (30.61%) of these genomes, respectively, indicating that 30–40% of *B. pseudomallei* genomes contained DNA similar to either vB_HM387 or vB_HM795 DNA, with vB_HM387-like DNA being more prevalent. Furthermore, 28/98 (28.57%) *B. pseudomallei* strains carried both vB_HM387-like and vB_HM795-like prophage DNA in their genomes. Taken together, hemoculture-isolated phages vB_HM387 and vB_HM795 are lysogenic phages that are highly similar to prophages harbored in the genomes of several *B. pseudomallei* strains that had been deposited in the NCBI database. Furthermore, our data indicated that some *B. pseudomallei* strains carried both vB_HM387-like and vB_HM795-like prophages in their genomes.

## Discussion

Most bacterial genomes deposited in public databases contain prophages, often constituting a sizable part of the total bacterial DNA. Prophage DNA can constitute up to 10–20% of the entire bacterial genome (Casjens, 2003), where one bacterial genome can carry more than one prophage. For example, *E. coli* O157:H7 strain Sakai harbors 18 prophages (approximately 16% of the total bacterial genome), which may be the highest number of prophages per genome in *E. coli* strains (Asadulghani et al., 2009). Similarly, there are several reports about prophage sequences in the genome of *B. pseudomallei* (Holden et al., 2004). Ronning et al. (2010) showed that 91% of *B. pseudomallei* strains carry at least one prophage. These studies also highlighted the significance of phages in conferring virulence genes on the bacterial host. However, the isolation of the induced prophage from blood collected from melioidosis patients has not been reported.

In this study, 12 phages were isolated from 40 hemoculture-positive samples (30.0% isolation rate) using *B. thailandensis* strains E174 and E332 as propagation hosts. The hemoculture bottles were kept in the refrigerator for a varying time ranging from 4 to 63 days before phage isolation, and it is likely that some of the phages may have been inactivated during storage. Furthermore, it is possible that not all the *B. pseudomallei* phages present in the hemocultures could form plaques on the *B. thailandensis* strains used in this study. Further studies to optimize isolation conditions, such as using additional *Burkholderia* host strains, will likely enhance the phage isolation rate.

Several factors have been reported to trigger prophage induction, including oxidative stress, osmotic stress, antibiotics, and elevated temperature (Filipiak et al., 2020; Bruneaux et al., 2022). We propose that reactive oxygen species (ROS) released in the human blood during *B. pseudomallei* infection and temperature at 37°C might be the possible factors responsible for prophage release from *B. pseudomallei* into the blood, leading to the detection of free phages from the hemoculture bottle. This hypothesis was supported by our investigation that *B. thailandensis*-spiked human venous blood incubated at room temperature showed the induction of free phage particles from the bacteria (Supplementary material). In addition, culturing of *B. thailandensis* in LB broth at 37°C revealed the induced prophage in the culture medium (Kanokudomsin et al., 2023). Taken together, these results suggest that components in blood and hemoculture, including ROS and/or temperature at 37°C, may be possible factors that induce prophage induction. However, the possibility of the spontaneous release of prophage from the bacteria without a specific inducer cannot be ruled out. The latter suggestion was supported by previous reports that some prophages could be spontaneously induced from *Ralstonia solanacearum* (Ahmad et al., 2017), Shiga toxin-producing *E. coli* (Loś et al., 2009; Shimizu et al., 2009), and *Streptococcus pneumoniae* (Carrolo et al., 2010) without specific induction.

In the human body, phages are found in many parts, including the skin, oral cavity, lungs, gut, and urinary tract (Barr, 2017). *E. faecalis* phages can be isolated from healthy human saliva with an isolation rate of 9.8% (Bachrach et al., 2003; Nascimento et al., 2021). *S. epidermidis* phages could be isolated from human skin, with an isolation rate of 46% (Valente et al., 2021). We found from this study that *Burkholderia*-free phage particles could be found in the blood circulation of melioidosis patients. This significant discovery is corroborated by Haddock et al. (2023), who observed *E. coli* phage DNA in the blood of sepsis patients.

Of note, prophage induction from the *B. pseudomallei* genome should lead to bacterial lysis. It has been reported that the numbers of *B. pseudomallei* in melioidosis patients’ blood can be as low as 1 CFU/mL (Wuthiekanun et al., 2007). The low concentration of *B. pseudomallei* detected in melioidosis patients may result from the killing of bacteria by the immune system, but also from the mechanism we propose, which has largely been ignored–that of lysis of bacteria following prophage induction. This suggestion was supported by a previous study where the presence of phages in clinical samples could interfere with bacteria detection because phages can destroy bacterial cells (Navarro and Muniesa, 2017) or that phages may be released if bacteria are intracellular but may still be released and circulate in the blood (Shan et al., 2021).

Two blood-isolated phages (vB_HM387 and vB_HM795) that gave different plaque morphologies were selected for further characterization. Both were identified as Myoviruses in the order of Caudovirales. At present, the most well-known *Burkholderia* phages are Myoviruses. For example, prophages induced from clinical strains of *B. pseudomallei* include phiK96243, phi52237, phiE12-2, and phi644-1 (Ronning et al., 2010). Six *B. pseudomallei* phages isolated from soil samples were also myoviruses (Yordpratum et al., 2011). Only our previous studies reported the isolation of *B. pseudomallei* phages from the soil samples that are podoviruses (Gatedee et al., 2011; Withatanung et al., 2016).

*B. pseudomallei* phages vB_HM387 and vB_HM795 could both infect *B. thailandensis*, but not other related species of *Burkholderia* or Gram-negative bacterial strains. The cross-infection of *B. pseudomallei* phages against *B. thailandensis* suggests that phages may impact the abundance of *B. pseudomallei* and *B. thailandensis* that co-exist in the same environment. Additionally, vB_HM795 showed higher capability than vB_HM387 to infect *B. thailandensis* that expresses a *B. pseudomallei*-like capsule, as is observed in BTCV strains. To infect bacterial cells, phages can bind to many bacterial structures, including the capsule, which also serves as a receptor for phage binding (Bertozzi Silva et al., 2016). The major difference between *B. thailandensis* and BTCV is that BTCV expresses a capsular polysaccharide that is highly similar to the *B. pseudomallei* capsule (Sim et al., 2010). The receptors on the bacterial cell surface responsible for vB_HM795 binding leading to infection may be capsular polysaccharides, whereas access to a receptor for vB_HM387 may be obstructed by the presence of a capsule in the BTCV strains. Another possibility that vB_HM387 could not infect the BTCV strains may be due to superinfection (Bondy-Denomy et al., 2016). The BTCV strains might be carrying a phage that is similar to phage vB_HM387, resulting in preventing infection of the BTCV strains.

In human blood circulation under physiological conditions without pathological states, pH ranges from 7.35 to 7.45 (Hopkins et al., 2022), and acidosis (pH <7.3) indicates poor prognosis in sepsis (Siddiqui et al., 2021), whereas pH levels in hemoculture bottles range from 6.94 to 7.26 (Welch et al., 1984). Due to the wide range of pH tolerances of phages vB_HM387 and vB_HM795, it is suggested that both phages could survive in a diverse pH environment, including human blood and hemoculture conditions. Additionally, both phages are able to survive at a physiological human body temperature of 37°C and during moderate fever from infection when the temperature is higher than 37°C. In severe fever (hyperthermia), body temperature can rise to greater than 41°C (Balli et al., 2023). Soil-isolated podovirus vB_BpP_HN01 specific to *B. pseudomallei* was found to be stable at a wide range of temperatures (24, 37, 40, 50, and 60°C) and pH values (3–12; Wang et al., 2022). When compared to the soil-isolated phage, the hemoculture-isolated phage showed less stability. This may be due to the fact that environment-adapted phages have to adapt themselves for survival in various environmental conditions.

The whole genomes of these two phages were also analyzed. The presence of TA genes in the phage vB_HM387 genome could potentially enhance antibiotic resistance and the virulence of the bacterial host (Hayes, 2003). This study revealed that phage vB_HM387 is a temperate phage, and the presence of TA (toxin–antitoxin) genes in its genome could potentially enhance antibiotic resistance and virulence in the bacterial host. This is because the *B. pseudomallei* RelE/B system (a homolog of our identified TA genes), a member of the type II toxin–antitoxin system, has been reported to contribute to antibiotic tolerance and treatment failure in melioidosis patients (Butt et al., 2013). The proposed mechanism involves promoting persister cell formation, which is dormant and can withstand stress conditions, aiding bacterial survival (Zou et al., 2022). Additionally, the RelE/B system is known to play a role in biofilm formation, resulting in enhanced intracellular survival in microorganisms like *Vibrio cholerae* and *Burkholderia cenocepacia* (Van Acker et al., 2014; Wang et al., 2015).

Additionally, *vapE* gene encoding the virulence-associated protein E was found in the phage vB_HM795 genome. Previously, the *vapE* gene has been found in pathogenicity islands (PIs) of *S. aureus* (Yarwood et al., 2002) and has a role in the virulence and pathogenesis of *S. suis* (Juhas et al., 2009). The *vapE* gene of *B. pseudomallei* was identified in the genome of strain 1026b, isolated from a patient with septicemic melioidosis. It is believed that the *vapE* gene’s presence in the *B. pseudomallei* genome is due to prophage integration (Johnson et al., 2015). Notably, the *vapE* gene is not found in all the *B. pseudomallei* strains, indicating it is not evolutionarily conserved and is likely acquired through horizontal gene transfer (Wong et al., 2022). This evidence suggests that phage vB_HM795, which carries the *vapE* gene, could enhance the virulence of its host *via* horizontal gene transfer. This theory is bolstered by the fact that prophages can promote the dissemination of genetic traits, aiding in the evolution and adaptation of bacterial pathogens (Brüssow et al., 2004). Nevertheless, the specific role of phage vB_HM795 in *B. pseudomallei* virulence warrants further study.

Phage vB_HM387 shows high similarity to other well-characterized *B. pseudomallei* phages deposited in the GenBank database, while phage vB_HM795 is a novel prophage. Moreover, different genes among phages vB_HM387 and other phages were usually found near the integrase, called “hotspots for the integration region.” This region was likely acquired by recombination between prophages and bacterial genomes or a horizontal gene transfer process (Hendrix et al., 2000). Searching the NCBI database using the BLAST algorithm revealed that phage vB_HM387 shows high sequence similarity to fragments of genomic DNA from various *B. pseudomallei* strains. These results indicated that the phage vB_HM387 can be lysogenized commonly across *B. pseudomallei* chromosomes isolated from various countries. While phage vB_HM795 is novel and shows high identity to various *B. pseudomallei* strains, it showed lower similarity to *B. pseudomallei* Thailand isolates.

In this study, we demonstrated the presence of *B. pseudomallei*-free phages, vB_HM387 and vB_HM795, in the hemoculture of melioidosis patients. Genome sequence analysis revealed that *B. pseudomallei* genomes contained either prophage vB_HM387 or vB_HM795, or both of them. Both prophages carried potential bacterial virulence genes in their genomes, suggesting the possibility that both phages can contribute to their host’s virulence by providing virulence genes via a horizontal gene transfer. Due to the presence of *Burkholderia* phages in the hemocultures of melioidosis patients, it is suggested that these phages are also likely to be induced and present in the circulation during infection *in vivo*. We suggest that the detection of phage genomic DNA, directly from the blood, can be a novel approach to improving the diagnosis of melioidosis. PCR targeting *Burkholderia*-specific phages may provide a significant sensitivity advantage compared to the currently used tests to detect *B. pseudomallei* in clinical samples. This hypothesis is based on the consideration that no *B. pseudomallei*-specific phages should be present in the blood of non-infected individuals. Furthermore, single prophage genomes could generate hundreds of phages released from a single bacterium. This strategy of phage-based detection assays would likely improve the sensitivity of the diagnostic test. The development of PCR targeting *Burkholderia* phages to improve the diagnosis of melioidosis is currently under investigation in our laboratory.

## Data availability statement

The datasets presented in this study can be found in online repositories. The names of the repository/repositories and accession numbers can be found at: https://www.ncbi.nlm.nih.gov/, OR990504; https://www.ncbi.nlm.nih.gov/, OR990505.

## Ethics statement

The studies involving humans were approved by Khon Kaen University Ethics Committee for Human Research, Faculty of Medicine, Khon Kaen University. The studies were conducted in accordance with the local legislation and institutional requirements. The ethics committee/institutional review board waived the requirement of written informed consent for participation from the participants or the participants’ legal guardians/next of kin because all hemoculture samples were leftover specimens taken from Srinagarind Hospital, Faculty of Medicine, Khon Kaen University. Retrieved of patients’ hemoculture samples were reviewed and approved by Khon Kaen University Ethics Committee for Human Research based on the Declaration of Helsinki and the ICH Good Clinical Practice Guidelines (approval number: HE651180). In addition, this research project was approved by the Institutional Biosafety Committee- IBC, Faculty of Medicine Siriraj Hospital, and Faculty of Tropical Medicine, Mahidol University. The approval certificate number are SI 2022–013 and MU 2023-002.

## Author contributions

PW: Conceptualization, Formal analysis, Investigation, Methodology, Writing – original draft, Writing – review & editing, Validation. SJ: Investigation, Writing – review & editing. MV: Investigation, Writing – review & editing. VM: Investigation, Writing – original draft. SC: Resources, Writing – review & editing. DB: Writing – review & editing, Investigation. VW: Resources, Writing – review & editing. EG: Formal analysis, Writing – review & editing. MC: Writing – review & editing. OG: Writing – review & editing, Methodology, Supervision. SK: Writing – original draft, Formal analysis, Conceptualization, Supervision, Writing – review & editing, Methodology.

## Footnotes

^1^ http://www.ncbi.nlm.nih.gov/BLAST
