# Supplementary material for: Induced Burkholderia prophages detected from the hemoculture: a biomarker for Burkholderia pseudomallei infection
Source: Front Microbiol. 2024 Apr 2;15:1361121. doi: 10.3389/fmicb.2024.1361121 (PMC11022660; doi:10.3389/fmicb.2024.1361121)
Supplement: Supplementary file 1 [file Data_Sheet_1.docx]

Supplemental Material

**Induced *Burkholderia* prophages detected from the hemoculture: a biomarker for *B. pseudomallei* infection**

^1^Patoo Withatanung, ^1^Sujintana Janesomboon, ^2^Muthita Vanaporn, ^1^Veerachat Muangsombut, ^3^Sorujsiri Charoensudjai, ^4^Dave J. Baker, ^5^Vanaporn Wuthiekanun, ^6^Edouard E. Galyov, ^6^Martha R.J. Clokie, ^7*^Ozan Gundogdu, ^1*^Sunee Korbsrisate

*** Correspondences:** Sunee Korbsrisate (sunee.kor@mahidol.edu) and Ozan Gundogdu (ozan.gundogdu@lshtm.ac.uk).

***B. thailandensis* spiked-human venous blood**

To investigate if human blood triggers prophage release from bacterial genomes, *B. thailandensis* was spiked into fresh normal human venous blood and PBS, followed by assessing viable bacteria and free phages. We found that free phages were detected in *B. thailandensis*-spiked human venous blood, but no free phages were detected in *B. thailandensis*-spiked PBS (Supplemental material). Additionally, after incubation, no viable *B. thailandensis* was detected in *B. thailandensis*-spiked human venous blood, but *B. thailandensis* remained viable in PBS. These findings imply that *Burkholderia* prophages could be induced by interactions of bacteria with human blood.

**Supplemental Table 1** Numbers of viable bacteria and free phages after incubating with human venous blood.

| Conditions | Viable bacteria  (CFU/ml) | Viable phages  (PFU/ml) |
| --- | --- | --- |
| Normal human venous blood (5 ml) + PBS | 0 | 0 |
| Normal human venous blood (5 ml) +  *B. thailandensis* (10^4^ CFU) | 0 | 2.3 ± 1.5 (×10^3^) |
| PBS + *B. thailandensis* (10^4^ CFU) | 2.7 ± 0.6 (×10^4^) | 0 |
